# Supplementary material for: A Wearable Infrared Sensor for Detecting Non-ST Segment Elevation Acute Coronary Syndromes
Source: Life (Basel). 2026 Jul 13;16(7):1155. doi: 10.3390/life16071155 (PMC13413182; doi:10.3390/life16071155)
Supplement: Supplementary file 1 [file life-16-01155-s001.zip › life-4389248-supplementary.pdf]

Supplementary Material

I. Machine-learning model development

<u>*Feature engineering and preprocessing*</u>

From each Infrasensor time series, we derived a structured feature set comprising statistical descriptors (mean, median, standard deviation, variance, range, interquartile range, skewness, kurtosis), percentiles (1st, 5th, 10th, 25th, 75th, 90th, 95th and 99th) and median absolute deviation. Time-domain energy and dynamics were summarized using root mean square (RMS), total energy, signal power, linear trend coefficients, mean change, mean absolute change and autocorrelation at lags 1 and 2. Waveform morphology was characterized by zero-crossing rate and peak-to-peak amplitude. Frequency-domain features were obtained from the magnitude spectrum of the fast Fourier transform (mean, standard deviation, minimum and maximum) and from Welch power spectral density estimates (mean, standard deviation, minimum and maximum). Complexity was quantified using Shannon entropy and unique value counts. Time–frequency structure was captured via short-time Fourier transform (STFT) spectrograms, from which mean, standard deviation, minimum and maximum values across time and frequency were extracted. Long-term dependence was summarized using the Hurst exponent. Features with near-zero variance or excessive missingness were excluded; remaining features were imputed (median within training folds) and scaled as needed.

<u>*Candidate models and hyperparameter optimization*</u>

We evaluated multiple classifiers: logistic regression, support vector machines (SVM), random forests, multilayer perceptrons (MLP), LightGBM and XGBoost gradient-boosting models and a stacked ensemble combining LightGBM and XGBoost. Hyperparameters for each model family were tuned using randomized search with nested cross-validation. For LightGBM and XGBoost, the search space included number of estimators, learning rate, maximum tree depth, minimum child samples, subsample ratio and column sampling rate. For tree-based models, class imbalance was addressed via class weights (positive:negative ≈ 5:1). Early stopping rounds were used for boosting models based on validation loss. All model development was implemented in Python 3.11.5 (Python Software Foundation), using scikit-learn, LightGBM and XGBoost libraries.

<u>*Stacked Ensemble Model*</u>

For the stacked ensemble, LightGBM and XGBoost base learners were first trained independently on the full feature set using optimized hyperparameters. Out-of-fold predicted probabilities from each base learner were then used as two-dimensional meta-features to train a gradient-boosting meta-learner, which produced the final

prediction. This stacking strategy enabled a dual ensemble with controllable weighting and complementary inductive biases, and was evaluated within a leave-one-cohort-out cross-validation scheme.

Final three-stage LightGBM ensemble

The final deployed architecture, described in the main text, consists of: (i) a Stage-1 LightGBM feature-selection model used to rank and select features; (ii) two Stage-2 LightGBM base classifiers with complementary configurations—Model 1 and Model 2; and (iii) Stage-3 ensemble aggregation, in which calibrated probabilities from the two models are averaged to obtain the final probability estimate, with a nominal decision threshold of 0.5.

Supplemental Table S1. List of hyperparameters for model development

Stage	Model / Component	Hyperparameter / Setting	Value
Stage 1 – Feature selection	LightGBM feature-selection model	n_estimators	550
		learning_rate	0.05
		num_leaves	32
		max_depth	−1 (unlimited)
		min_child_samples	22
		subsample	0.8
		colsample_bytree	0.8
		random_state	41
Stage 2 – Base classifier 1	LightGBM Classifier (Model 1)	n_estimators	300
		learning_rate	0.05
		num_leaves	32
		max_depth	−1
		min_child_samples	23
		subsample	0.8
		colsample_bytree	0.5
		class_weight	{1: 5, 0: 1}
		random_state	31
		Calibration method	Sigmoid (Platt scaling, CalibratedClassifierCV)
Stage 2 – Base classifier 2	LightGBM Classifier (Model 2)	n_estimators	620
		learning_rate	0.04
		num_leaves	48
		max_depth	−1
		min_child_samples	20
		subsample	0.8
		colsample_bytree	0.6
		class_weight	{1: 5, 0: 1}
		random_state	99
		Calibration method	Sigmoid (Platt scaling, CalibratedClassifierCV)
Stage 3 – Ensemble aggregation	Probability averaging	Aggregation formula	(p1 + p2)/2
		Decision threshold	0.5
		Class distribution handling	Imbalanced, 5:1 weighting for positive class in base models

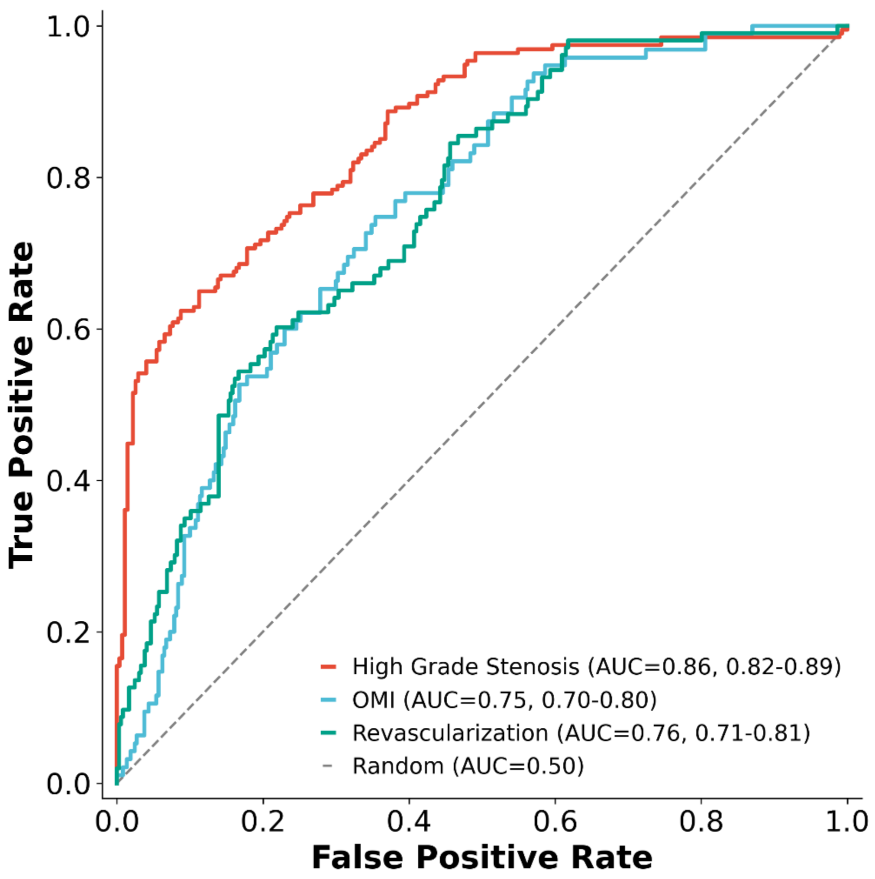

Supplemental Figure S1. Receiver operating characteristic curves for the High Grade Stenosis, Occlusion MI, and Revascularization models.

Supplemental Table S2. Classification performance for the High-Grade Stenosis, Occlusion MI, and Revascularization models.

Model	Events	Sensitivity (%)	Specificity (%)	PPV (%)	NPV (%)	Accuracy (%)	F1-Score (%)	AUC (95% CI)
High Grade Stenosis	194	64.4	88.7	80.1	77.9	78.7	71.4	0.86 (0.82-0.89)
Occlusion MI	95	62.1	74.6	38.6	88.5	72.0	47.6	0.75 (0.70-0.80)
Revascularization	109	62.1	74.9	41.0	87.5	72.1	49.4	0.76 (0.71-0.81)

II. Feature Importance Analysis

Supplemental Figure S2 displays the SHAP beeswarm plot visualizing feature importance and directional impact for the Infrasensor model. Features are ranked by importance (top = most influential), with each point representing a patient. Horizontal SHAP values indicate feature impact: positive values (right of zero) increase predicted probability of obstructive coronary disease, negative values decrease it. Color represents feature magnitude (yellow/green = high, purple = low). Shannon entropy demonstrated highest importance, with elevated values consistently associated with positive SHAP values, indicating greater signal complexity predicts obstruction. Similarly, STFT median showed strong positive associations when high. Conversely, temporal mean absolute change exhibited inverse relationships, with high values reducing obstruction probability. The horizontal spread indicates discriminative contribution—wider distributions (Shannon entropy, STFT median) distinguish obstructive from non-obstructive cases more effectively. Overall, the model integrates complementary features across spectral, temporal, and complexity domains, with spectral and complexity metrics providing the strongest discriminative power.

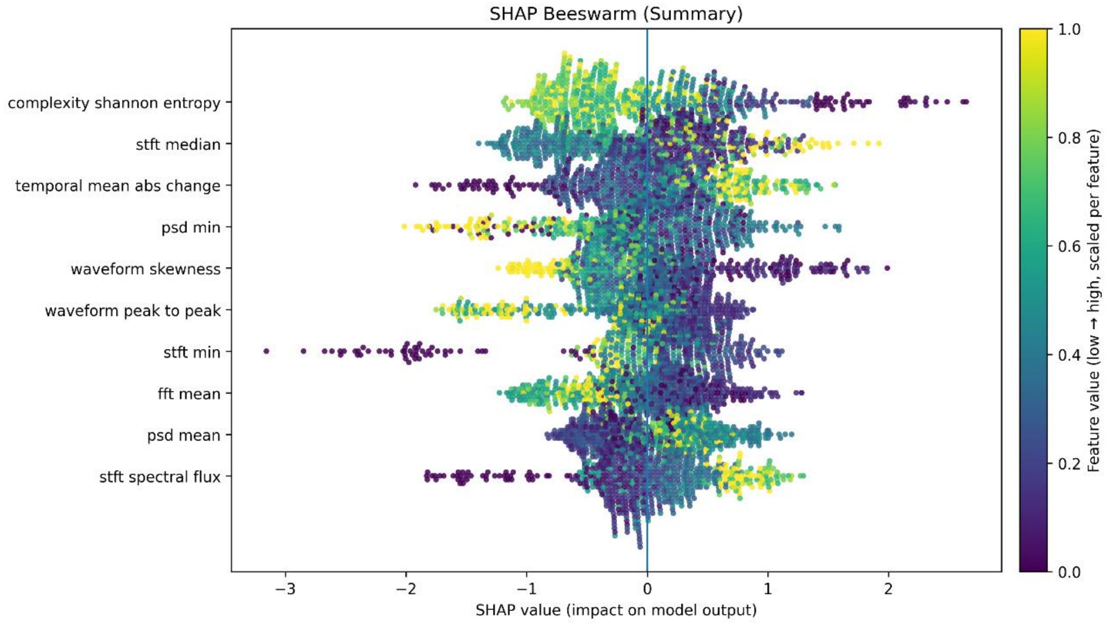

Supplemental Figure S2. Global Feature Importance and Directional Impact on Infrasensor Model Predictions Using SHAP Analysis

Supplemental Table S3. Clinical Characteristics and Biomarker Profiles of Patients with ACS Stratified by Prediction Model Classification (True Positive, True Negative, False Positive, False Negative)

Variable	FN (n=83)	FP (n=24)	TN (n=230)	TP (n=132)
**Demographics**				
Age, years	61 (55-69)	51 (46-64)*	54 (46-63)	61 (55-68)#
Male, n (%)	61 (75.3)	13 (56.5)	148 (64.6)	83 (70.3)
BMI ≥30 kg/m2, n (%)	11 (13.3)	5 (20.8)	46 (20.0)	14 (10.6)#
**Risk Factors**				
Diabetes, n (%)	38 (47.5)	10 (43.5)	70 (32.3)	67 (57.3)#
Hypertension, n (%)	47 (58.8)	17 (73.9)	124 (56.4)	86 (73.5)#
Smoker/Ex-smoker, n (%)	16 (20.5)	2 (8.7)	43 (20.1)	18 (15.5)
CKD, n (%)	1 (1.4)	0 (0.0)	4 (1.9)	2 (1.7)
Hyper-cholestrolemia, n (%)	13 (16.2)	4 (17.4)	24 (11.1)	15 (12.9)
Known history of CAD, n (%)	33 (41.8)	7 (30.4)	71 (34.0)	47 (39.8)
**Clinical Presentation**				
NSTEMI, n (%)	45 (55.6)	3 (12.5)*	29 (12.6)	62 (53.4)#
MINOCA, n (%)	0 (0.0)	3 (12.5)*	19 (8.3)	0 (0.0)#
Unstable Angina, n (%)	35 (43.2)	5 (20.8)	36 (15.7)	54 (46.6)#
INOCA, n (%)	1 (1.2)	0 (0.0)	15 (6.5)	0 (0.0)#
**CAG**				
OMI, n (%)	31 (37.8)	1 (4.2)*	5 (2.2)	58 (45.0)#
Severe Obstructive CAD, n (%)	80 (96.4)	1 (4.2)*	3 (1.3)	131 (99.2)#
**Treatment**				
PCI, n (%)	33 (39.8)	0 (0.0)*	1 (0.4)	56 (42.4)#
CABG, n (%)	5 (6.0)	0 (0.0)	0 (0.0)	8 (6.1)#
**Laboratory Results**				
Elevated hs-cTn, n (%)	45 (55.6)	3 (13.0)*	29 (12.7)	62 (53.4)#
hs-cTn > 200 times normal, n (%)	12 (14.8)	1 (4.3)	5 (2.2)	20 (16.9)#
NT-proBNP (pg/ml)	286 (141-904)	186 (78-341)*	170 (84-391)	397 (160-1000)#

Values are median (IQR) or n (%). Continuous variables compared using Mann-Whitney U or Kruskal-Wallis tests; categorical variables using chi-squared test. *p < 0.05 FP vs FN; #p < 0.05 TP vs TN. TP = true positive; TN = true negative; FP = false positive; FN = false negative. NSTEMI = non-ST elevation myocardial infarction; MINOCA = MI with non-obstructive coronary arteries; INOCA = ischemia with non-obstructive coronary arteries; OMI = old myocardial infarction; CAG = coronary angiography; PCI = percutaneous coronary intervention; CABG = coronary artery bypass grafting; hs-cTn = high-sensitivity cardiac troponin; NT-proBNP = N-terminal B-type natriuretic peptide; CKD = chronic kidney disease.

Supplemental Table S4. Complete reclassification pathways from HEART score to angiographic outcome for "rule-in" performance of Infrasensor

HEART Score	Infrasensor	Severe Coronary Obstruction	N	% of HEART Category
**Low**	High (n=11)	Obstructive	8	15.7%
		Non-obstructive	3	5.9%
	Low (n=40)	Obstructive	4	7.8%
		Non-obstructive	36	70.6%
**Low Subtotal**			**51**	**100.0%**
**Medium**	High (n=49)	Obstructive	36	18.6%
		Non-obstructive	13	6.7%
	Low (n=145)	Obstructive	27	13.9%
		Non-obstructive	118	60.8%
**Medium Subtotal**			**194**	**100.0%**
**High**	High (n=67)	Obstructive	63	48.1%
		Non-obstructive	4	3.1%
	Low (n=64)	Obstructive	33	25.2%
		Non-obstructive	31	23.7%
**High Subtotal**			**131**	**100.0%**

Supplemental Table S5. Complete Reclassification pathways from HEART Score to angiographic outcome for "rule-out" performance of Infrasensor

HEART Score	Infrasensor	Severe Coronary Obstruction	N	% of HEART Category
**Low (≤3)**	High (n=44)	Obstructive	23	35.9%
		Non-obstructive	21	32.8%
	Low (n=20)	Obstructive	2	3.1%
		Non-obstructive	18	28.1%
**Low Subtotal**			**64**	**100.0%**
**Medium (4-6)**	High (n=177)	Obstructive	123	50.6%
		Non-obstructive	54	22.2%
	Low (n=66)	Obstructive	0	0.0%
		Non-obstructive	66	27.2%
**Medium Subtotal**			**243**	**100.0%**
**High (≥7)**	High (n=156)	Obstructive	145	86.8%
		Non-obstructive	11	6.6%
	Low (n=11)	Obstructive	3	1.8%
		Non-obstructive	8	4.8%
**High Subtotal**			**167**	**100.0%**

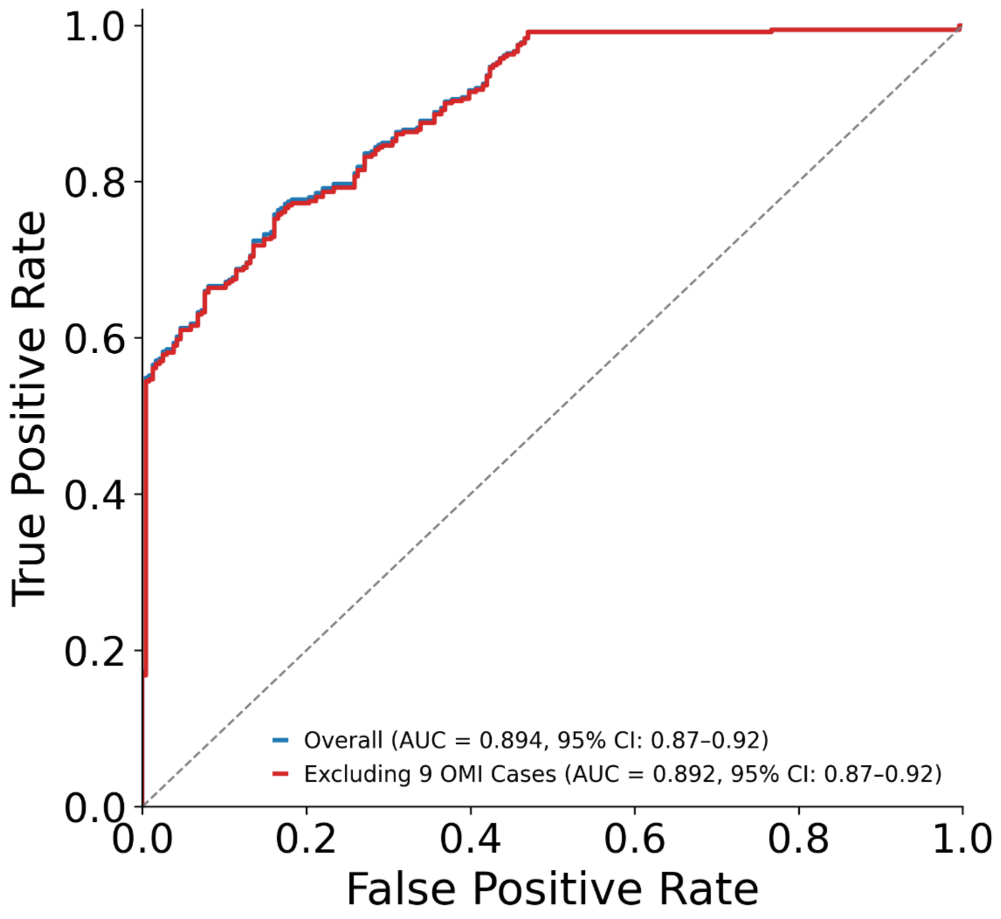

Supplemental Figure S3. Sensitivity Analysis Showing Robustness of Model Performance Even After Excluding OMI Patients with Biochemical Criteria.
